# Studies of the Sorption-Desorption of Pesticides from Cellulose-Based Derivative Nanocomposite Hydrogels

**DOI:** 10.3390/molecules29204932

**Published:** 2024-10-18

**Authors:** Fabrício C. Tanaka, Uilian G. Yonezawa, Marcia R. de Moura, Fauze A. Aouada

**Affiliations:** Hybrid Composites and Nanocomposites Group (GCNH), School of Engineering, São Paulo State University (Unesp), Ilha Solteira 15385-007, SP, Brazil; tanaka.fabricio@gmail.com (F.C.T.); uilianyonezawa@gmail.com (U.G.Y.); marcia.aouada@unesp.br (M.R.d.M.)

**Keywords:** hydrogel, cellulose derivatives, zeolite, herbicides, agricultural area

## Abstract

This study analyzed the effect of cellulose derivatives, namely methylcellulose (MC) and carboxymethylcellulose (CMC), on the stability of zeolite in a polymeric solution that would synthesize a three-dimensional network of poly(methacrylic acid)-co-polyacrylamide (PMAA-co-PAAm). Additionally, it investigated the effect of pH on the release of paraquat (PQ) and difenzoquat (DFZ) herbicides. Similar to previous studies with hydrogels containing CMC, the presence of bi and trivalent salts, such as Ca^+2^ and Al^+3^, also drastically reduced their swelling degree from 6.7 g/g in NaCl (0.15 mol·L^−1^) to 2.1 g/g in an AlCl_3_ solution (0.15 mol·L^−1^) for the MC nanocomposite. The viscosity results may suggest that the formation of a polysaccharide-zeolite complex contributed to the zeolite stabilization. As for the adsorption results, all samples adsorbed practically the entire concentration of both herbicides in an aqueous solution. Finally, it was also observed that the valence of the salts and molecular weight of the herbicide affect the release process, where DFZ was the herbicide with the highest concentration released. Both nanostructured hydrogels with CMC and MC exhibited lower release at pH = 7.0. These results demonstrated that a more in-depth evaluation of the phenomena involved in the application of these materials in controlled-release systems could help mitigate the impact caused by pesticides.

## 1. Introduction

In some countries, where the economy is highly dependent on agricultural production—such as Brazil, being one of the world’s largest producers of various raw materials and agricultural food products, including soybeans, corn, cotton, oranges, and coffee—the use of technologies to enhance the country’s agricultural yield efficiency, such as pesticide application, is widespread among rural producers [1]. However, the indiscriminate use of these chemical compounds could lead to severe damage to the environment and human health. According to Paumgartten [2], studies carried out in Brazil have shown a correlation between pesticide contamination and an increase in some diseases or dysfunctions, such as breast cancer, colon cancer, non-Hodgkin lymphoma, skin melanoma, depressive disorders, and male fertility.

Herbicides are a type of agrochemical used for weed management and control, and they contribute significantly to water pollution caused by pesticides worldwide [3]. Paraquat (1,1-dimethyl-4,4-bipyridinium ion) (PQ) is a nitrogenous, fast-acting, non-selective herbicide; it is one of the most widely used herbicides globally and is also associated with several reports of poisoning attributed to these compounds [4,5]. Difenzoquat methyl sulfate (DFQ) is another herbicide that belongs to the quaternary ammonium salts group and is a selective herbicide used for post-emergence control of wild oats in cereal crops [6]. It causes severe skin and eye irritations as well as other health problems due to its ability to affect several organs [7]. Due to the high solubility of both herbicides in aquatic environments, these molecules have the potential to have significant impacts on human health, particularly in populations living near areas where these herbicides are applied [8].

One promising strategy widely explored in the literature is the use of polymeric matrices capable of incorporating agrochemical molecules, enabling their application in removal or controlled release systems [9,10,11]. Due to their ease of processing and chemical modification, a wide range of matrices can be developed to tailor their physical and mechanical properties, making them suitable for various applications in agricultural systems [12,13].

Within the class of polymeric materials, there are 3D networks known as hydrogels, which can absorb water, solutions, and fluids. These hydrogels have been extensively studied and reported in the literature for various applications, including the remediation of water contaminated by herbicides [14,15,16,17]. When immersed in an aqueous medium, the thermodynamics of a hydrogel with ionic groups in its composition reaching an equilibrium state are described by Ritger and Peppas [18]. Equation 1 illustrates the Gibbs free energy involved in this process.

(1) $${∆G}_{total}={∆G}_{elastic}+{∆G}_{mixing}+{∆G}_{ionic}$$

where Δ*G_elastic_* represents the contribution of opposing forces that reduce chain elasticity within the hydrogel, and Δ*G_mixing_* and Δ*G_ionic_* denote the polymer’s affinity with the surrounding fluid molecules and the contribution of the ionic nature in the polymer network, respectively.

The use of co-polymer networks is another strategy that can be employed to produce novel hydrogels with improved properties [9,11]. According to studies conducted by Mittal et al. [19,20], matrices based on poly(methacrylic acid)-co-polyacrylamide (PMAA-co-PAAm) can generate biodegradable hydrogels with promising properties for removing cationic dyes from solutions. The incorporation of polysaccharides, such as cellulose derivatives, can enhance certain hydrogel properties, including hydrophilicity, biodegradability, and biocompatibility. Additionally, the presence of polysaccharides in the hydrogel composition can reduce the production costs due to the abundance of these raw materials in nature.

Another approach to enhancing swelling and improving sorption and desorption properties is the incorporation of non-toxic aluminum silicate nanostructures abundantly found in the environment, such as nanoclays and zeolites [21]. Zeolite, a hydrated aluminum silicate, consists of an overlay of the tetrahedral structures capable of forming various types of three-dimensional porous crystalline structures. These structures find extensive applications in purification, adsorption, and catalytic systems [22,23]. Several studies in the literature, including those conducted by Rashidzadeh et al. [24] and Sakar et al. [25] have reported that the addition of zeolite to their respective hydrogels developed by these authors leads to more controlled and slower desorption of fertilizers.

In previous studies conducted by us [9,11], novel hydrogel nanocomposites based on cellulose derivatives carboxymethylcellulose (CMC) and methylcellulose (MC) were developed. It was observed that the presence of zeolite in PMAA-co-PAAm hydrogel matrices with these polysaccharides increased the interaction forces with agrochemical inputs, such as fertilizers and the herbicide diquat. In one of these studies [26], a higher loading capacity of diquat molecules into an MC nanocomposite was reported. This observation was supported by the comparison of quantity of diquat adsorbed (mg) per gram of sample values (q_eq_), which were 8.02 mg·g^−1^ for the hydrogel and 9.66 mg·L^−1^ for the nanocomposite. Similar tendencies were also observed in other studies investigating the sorption of diquat by the hydrogel with CMC, with values of 8.30 mg·g^−1^ for hydrogel and 9.40 mg·L^−1^ for nanocomposite. The nanocomposite with lower q_eq_ presented a lower release of diquat compared to the hydrogel across all three different pH ratios studied. This behavior can be attributed to the enhanced interactions between the material and the herbicide. Based on these discussions, to further explore the potential of PMAA-co-PAAm hydrogels with cellulose derivatives (CMC or MC) and zeolite in agricultural systems, investigating other quaternary ammonium salt herbicides, such as paraquat and difenzoquat, could provide valuable insights for their application.

The present study aims to investigate the effect of cellulose derivatives, namely CMC and MC, as thickening agents in zeolite stabilization within the PMAA-co-PAAm synthesis solution. Additionally, the study analyzes the potential of these systems for the removal and release of paraquat and difenzoquat herbicides through sorption tests. The influence of pH on desorption properties was also studied. This research examines the effect of zeolite presence in the MC hydrogel structure on thermal properties, specifically thermal stability, using thermogravimetric analysis (TG). Moreover, the study explores the influence of saline ions on water absorption in the swelling medium. Previous works have discussed the changes induced by zeolite in CMC nanocomposites regarding thermal properties and the ionic force affecting the degree of swelling [9,11]. The novelty of this article lies in the investigation of the effect of polymer structures on the adsorption and release of different pesticides. By associating these data with results from other previously cited herbicides, it will be possible to discuss more promising mechanisms for utilizing these nanostructured hydrogels more efficiently in agriculture-oriented applications.

## 2. Results and Discussion

### 2.1. Apparent Viscosity (μ)

In previous studies conducted by the research group, it was observed that the addition of thickeners such as CMC not only increased the viscosity of the medium but also helped to stabilize zeolite in the polymeric solution during synthesis, thereby preventing the formation of precipitates [9]. To investigate the stability of zeolite in the polymerization solution and understand the interactions between the zeolite nanostructure and the hydrogel components, measurements of apparent viscosity and pH were performed (Table 1).

The viscosity of the polymeric solution was influenced by the amount of polysaccharide present or by the strength of the interaction forces between water molecules and the hydrophilic groups in the macromolecule. The higher viscosity of the CMC solution without zeolite (336.9 cP) can be attributed to its higher molecular mass. Due to the superior thickening properties of CMC, a lower amount of polysaccharide (0.75% *w*/*v* of CMC) was required compared to the 1.0% *w*/*v* of MC. Another factor is the higher hydrophilicity of carboxyl groups in CMC as compared to the methyl groups in MC. This phenomenon also explains the observed increase in viscosity in the CMC nanocomposite solution, which can be attributed to the formation of a zeolite-polysaccharide complex with higher molecular mass and superior thickening properties.

### 2.2. Thermal Analysis

#### Thermogravimetric Analysis (TG) and Differential Thermogravimetric Analysis (DTG)

Thermogravimetric analysis (TG) and derivative thermogravimetric (DTG) curves were utilized in this study to investigate the degradation temperatures of the matrix hydrogel and hydrogels with MC, as well as to explore the influence of zeolite on the thermal stability of these materials.

For the hydrogel without zeolite, a minor decline can be observed at approximately 38.6 °C to 163.2 °C, corresponding to a loss of 5.4% of the sample mass mainly, due to the loss of hydration water. Next, a small decline in the baseline is observed, starting after 163.2 °C, accompanied by a sharp drop until 271.7 °C. Within this temperature range, approximately 12.6% of the sample mass is lost, which can be associated with the loss of water and ammonia resulting from the anhydrization of COOH groups and the imidization of NH_2_ groups, respectively. Finally, the most significant drop in the curve occurs between 271.7 °C and 556.5 °C, indicating the decomposition of anhydride and imide groups formed during the previous thermal event, as well as the scission of the hydrogel main chain, resulting in the production of carbon dioxide and water [27,28,29,30].

**Figure 1:**
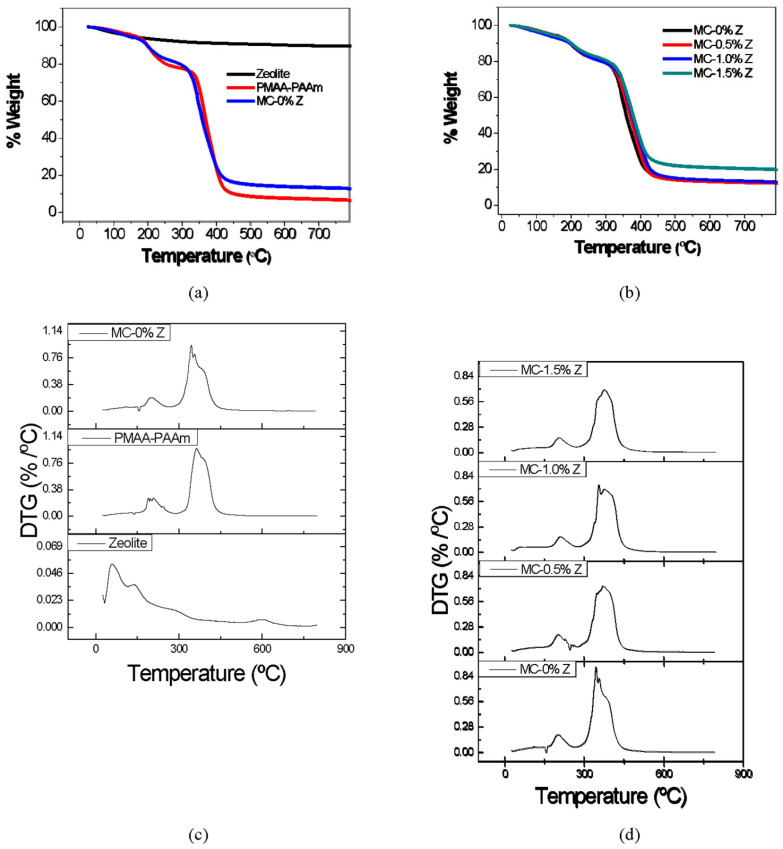
(**a**) TG curves of zeolite (in black), matrix hydrogel (in red), and hydrogel with MC (in blue). (**b**) TG curves of hydrogel with MC (in black) and its nanocomposites with 0.5%, 1.0%, and 1.5% *w*/*v* of zeolite (in red, blue, and green, respectively). (**c**,**d**) DTG curves obtained from (**a**,**b**), respectively.

The curves for the nanostructured hydrogels are represented in Figure 1b. It can be observed that there is no significant increase in the residual mass for samples with 0.5% and 1.0% zeolite, while the hydrogel with 1.5% zeolite shows a considerable increase of 21.2% in residual mass. However, in the third degradation event, related to the scission and depolymerization of hydrogel and polysaccharide chains observed at 258.2 °C and 572.2 °C for the hydrogel without zeolite, there is a reduction of 68.5% in mass loss. There is a tendency towards a reduction in the percentage of mass loss for hydrogels containing zeolite concentrations above 1.0% in the hydrogel structure. The mass loss in this temperature range for hydrogels with 0.5%, 1.0%, and 1.5% zeolite was 69.1%, 67.6%, and 62.4%, respectively. A reduction in the percentage of mass loss was also observed with increasing zeolite concentration in the second degradation event, occurring between 163.2 °C and 271.7 °C, with values of 12.6% for the hydrogel without zeolite. Within this temperature range, hydrogels with 0.5%, 1.0%, and 1.5% zeolite exhibited mass loss percentages of 12.3%, 11.8%, and 10.6%, respectively.

Regarding the initial mass loss associated with the removal of bound water occurring between 38 °C and 163.2 °C, the hydrogel without zeolite exhibited a loss of 5.4%. In the case of hydrogels with zeolite, an increase in mass loss was observed with higher zeolite concentrations, reaching 6.1%, 7.3%, and 6.6% for nanocomposites with 0.5%, 1.0%, and 1.5% zeolite, respectively. This increase in mass loss is attributed to the greater presence of bound water from zeolite within the matrix of the nanocomposites [30].

The DTG curves presented in Figure 1c,d were derived from the previously discussed TG curves. The black curve, representing only zeolite, showed a single significant mass loss between approximately 29.5 °C and 200.6 °C, with a peak at 57.9 °C. This temperature range is characteristic of hydration water loss, which is also observed in hydrogels. Beyond this range, zeolite exhibited a gradual and minor mass loss with increasing temperature, without any peaks corresponding to a high mass loss.

From Table 2, it was possible to observe an increase in the values of T_0_, T_f_, and T_max_ for 2° and 3° thermal events caused by increasing the concentration of zeolite, indicating that this structure enhanced the thermal stability of the materials. This can be attributed to the absorption of thermal energy, which helps break the interactions between the zeolite and the hydrophilic groups of the matrix. Additionally, a portion of the energy is adsorbed by the zeolite structure itself. The TG and DTG confirm that zeolite exhibits minimal mass loss within the temperature range examined.

Finally, the TG and DTG data, along with previously published results on FTIR, XRD, SEM, and EDX analyses, support the successful incorporation of zeolite into the hydrogel matrix based on cellulose derivatives (CMC and MC) and PMAA-co-PAAm networks.

### 2.3. Influence of Osmotic Force on the Swelling Degree Properties of MC Nanocomposites

**Figure 2:**
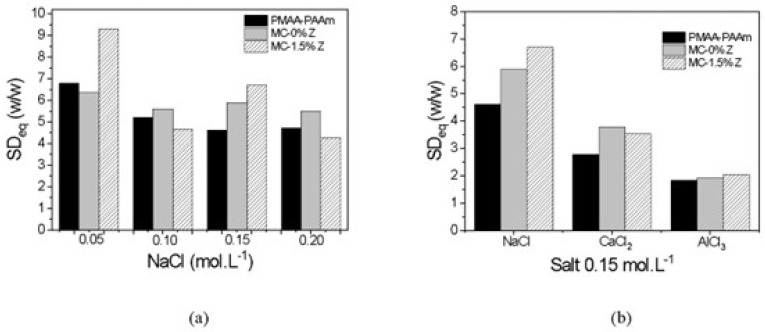
Bar graph of *SD_eq_* as a function of NaCl (**a**) and NaCl, CaCl_2_, or AlCl_3_ at 0.15 mol·L^−1^ (**b**) solution concentrations for the matrix hydrogels, MC-containing hydrogels, and their nanocomposites with 1.5% *w*/*v* of zeolite.

Figure 2a shows the equilibrium swelling degree (*SD_eq_*) of the matrix hydrogel, MC hydrogel, and MC–zeolite nanocomposite in different saline solutions (0.10, 0.15, or 0.20 mol·L^−1^ NaCl). The results indicate a general reduction in the swelling degree for all samples. However, the reduction was less significant for the MC hydrogel and zeolite hydrogel. Interestingly, the polysaccharide hydrogel exhibited higher swelling degree values at NaCl concentrations above 0.05 mol·L^−1^. Unlike the matrix hydrogel and CMC hydrogels, the presence of a methyl group in the MC hydrogel resulted in weaker intra- and intermolecular interactions, leading to less pronounced negative effects on water storage capacity caused by the presence of ions in the swelling medium. Notably, higher swelling degree values than those of the matrix hydrogel were observed in saline solutions with bi- and trivalent ions Ca^+2^ and Al^+3^, as shown in Figure 2b.

### 2.4. Herbicide Sorption

**Figure 3:**
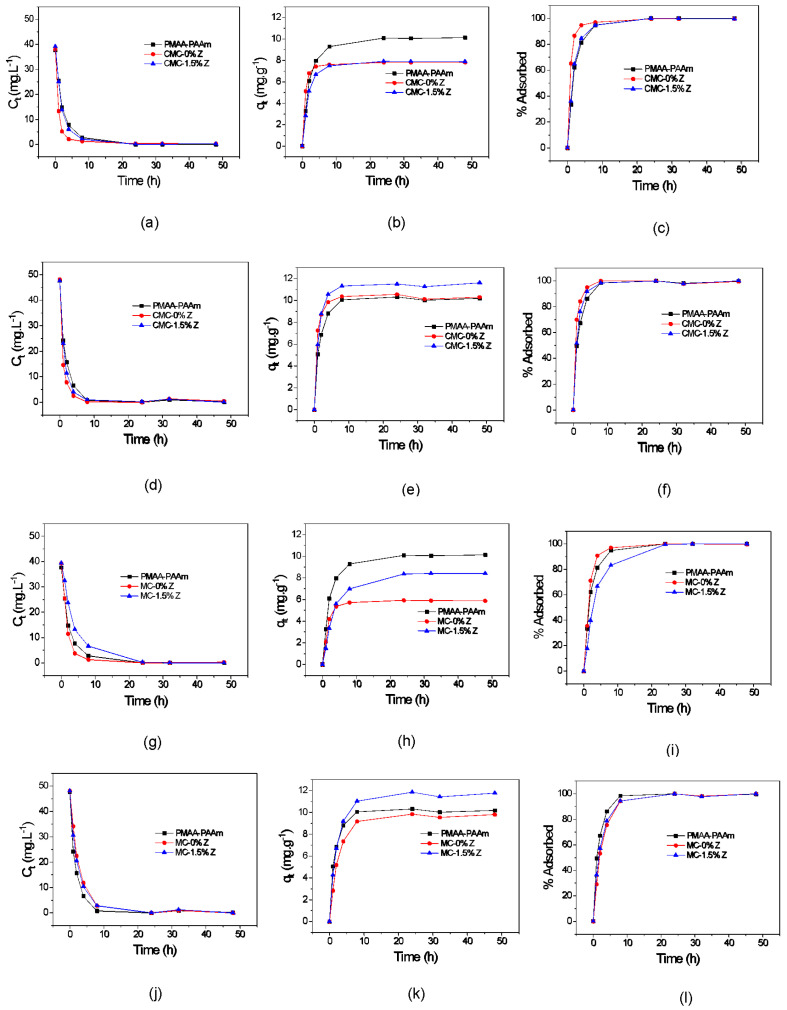
Kinetic curves of the matrix hydrogel, hydrogel with polysaccharide, and hydrogel nanocomposite with CMC concerning the concentration of adsorbed paraquat over time (**a**), adsorptive capacity (*q_eq_*) over time (**b**), and percentage adsorbed over time (**c**). Kinetic curves of the matrix hydrogel, hydrogel with polysaccharide, and hydrogel nanocomposite with CMC regarding the concentration of adsorbed difenzoquat over time (**d**), adsorptive capacity (*q_eq_*) over time (**e**), and percentage adsorbed over time (**f**). Kinetic curves of the matrix hydrogel, hydrogel with polysaccharide, and hydrogel nanocomposite with MC in relation to the concentration of adsorbed paraquat over time (**g**), adsorptive capacity (*q_eq_*) over time (**h**), and percentage adsorbed over time (**i**). Lastly, kinetic curves of the matrix hydrogel, hydrogel with polysaccharide, and hydrogel nanocomposite with MC concerning the concentration of adsorbed difenzoquat over time (**j**), adsorptive capacity (*q_eq_*) over time (**k**), and percentage adsorbed over time (**l**).

The kinetic curves of the adsorbed paraquat, adsorptive capacity, and percentage of herbicide adsorbed by the hydrogels and their respective nanocomposites are represented in Figure 3a–c. The same curves related to the difenzoquat are shown in Figure 3d–f. The results showed that the polysaccharide-based hydrogel without zeolite had faster sorption compared to the matrix and nanocomposite. The curves indicated that the CMC sample adsorbed almost all the paraquat and difenzoquat in the solutions within 8 h. The hydrogel matrix exhibited a slight change in herbicide adsorption, despite having a lower attraction magnitude due to the absence of a positive charge in its structure.

However, there was no reduction in the capacity to store difenzoquat compared to paraquat. The initial concentration of difenzoquat in the adsorption solution was higher, possibly influencing the result of difenzoquat adsorption per gram of hydrogel, leading to a value similar to that of paraquat. In summary, the incorporation of CMC and zeolite in the paraquat curves possibly increased adsorption, while in the difenzoquat curves, the addition of CMC and zeolite enhanced adsorptive capacity.

Unlike paraquat adsorption with CMC, hydrogels with MC showed slower kinetics (Figure 3g–i), possibly due to excessive interactions between hydrophilic groups and herbicide molecules, attributed to the higher polysaccharide content (0.75% *w*/*v* CMC vs. 1.0% *w*/*v* MC). However, unlike CMC hydrogels, zeolite presence in the MC hydrogel matrix delayed paraquat adsorption. The red curves in Figure 3h,k reveal a lower herbicide sorption capacity in the polysaccharide hydrogel compared to the matrix and nanocomposite. It is attributed to the less elastic polymer chains in the hydrogel with polysaccharide and weaker attraction forces compared to the nanocomposite, as zeolite charges are absent. Notably, zeolite addition enhanced the adsorptive capacity for both herbicides, particularly with difenzoquat, surpassing the hydrogel matrix performance.

### 2.5. Release Properties

To investigate the potential use of hydrogels and nanostructured hydrogels for the controlled release of herbicides in agriculture, experiments were conducted with two types of herbicides (paraquat and difenzoquat). Initially, herbicide release tests were carried out using distilled water as the medium, but no significant release of trapped agrochemical molecules was observed. However, when 0.1 mol·L^−1^ NaCl solutions with different pH ranges were used as the release medium, there was a noticeable increase in herbicide concentration over time.

**Figure 4:**
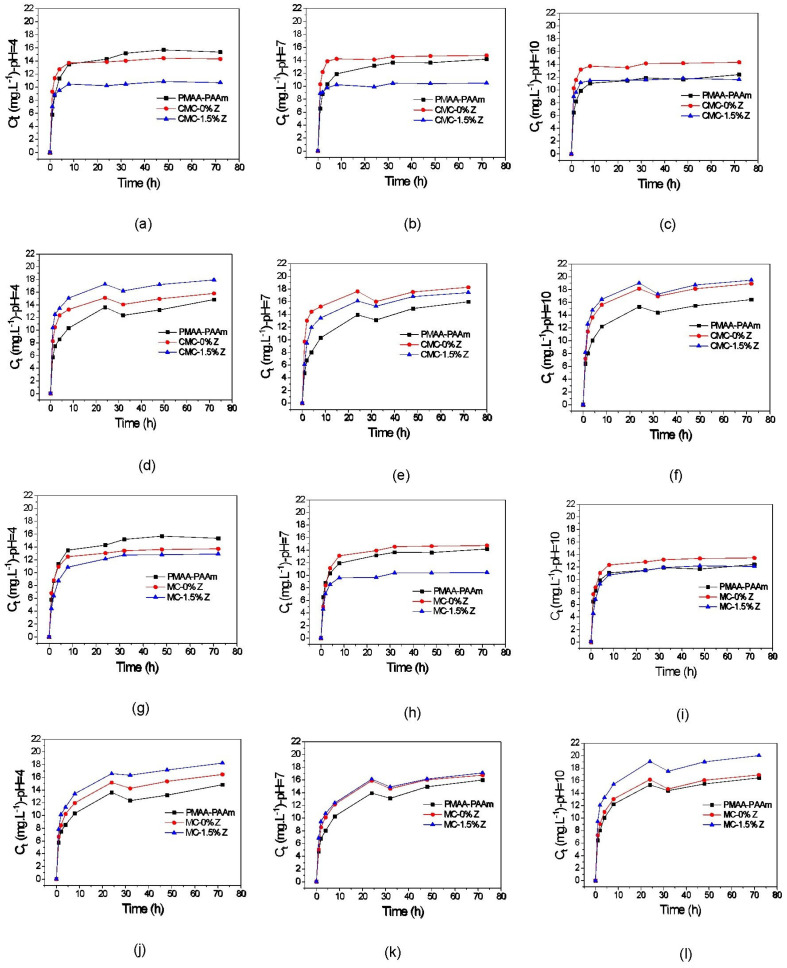
Kinetic release curves of the matrix hydrogel, hydrogel, and hydrogel nanocomposite with CMC, regarding the concentration of paraquat (pH 4 (**a**), 7 (**b**), and 10 (**c**)), and difenzoquat (pH 4 (**d**), 7 (**e**), and 10 (**f**)) desorbed over time. Kinetic release curves of the matrix hydrogel, hydrogel, and hydrogel nanocomposite with MC, regarding the concentration of paraquat (pH 4 (**g**), 7 (**h**), and 10 (**i**)) and difenzoquat (pH 4 (**j**), 7 (**k**), and 10 (**l**)) desorbed over time.

The results presented in Figure 4a–c, and Table 3 indicate that the hydrogel with CMC released a higher amount of paraquat (4.96 and 4.53 mg·g^−1^) compared to the matrix hydrogel (4.94 and 4.26 mg·g^−1^) and the nanostructured hydrogel (3.17 and 3.80 mg·g^−1^) for release media with pH 7.0 and 10. However, in an acidic medium (pH 4.0), the matrix hydrogel showed the highest amount of paraquat released. The diffusion capacity of Na^+^ ions into the hydrogel was influenced by the pH, with lower pH values favoring an increase in diffusion and release of paraquat. The presence of carboxyl groups contributed to these differences in release behavior. The hydrogel with CMC and the nanocomposite hydrogel showed higher paraquat release at higher pH values, whereas the matrix hydrogel performed better at lower pH values.

The release of difenzoquat, shown in Figure 4d–f, reached equilibrium within 24 h for the matrix hydrogel, hydrogel with CMC, and nanostructured hydrogel. The slower release of difenzoquat in the three pH ranges may be attributed to the stronger attraction between difenzoquat and the polymeric chains of the investigated hydrogels, as discussed earlier in this study in relation to diquat release curves by the matrix hydrogel, which exhibited a similar behavior due to the same phenomenon.

From Figure 4g–i, it is possible to see a rapid initial sorption rate for the matrix hydrogel and the hydrogels with MC within the first eight hours, followed by a gradual release of paraquat molecules. Beyond this period, no significant paraquat release is observed. The hydrogels with MC demonstrate a more controlled paraquat release compared to those with CMC due to the absence of carboxyl groups, reducing attractive forces and leading to slower diffusion and exchange of paraquat molecules. The addition of zeolite to the hydrogels with MC accelerates paraquat release, but an excessive formal charge can hinder it. The nanostructured hydrogel with MC exhibits higher paraquat release per gram of hydrogel compared to the hydrogel with MC in solutions with pH 4 and 10. The hydrogel with CMC releases a greater amount of paraquat per gram of hydrogel in all three pH ranges due to the greater contribution of carboxyl groups to diffusion and exchange, while the hydrogel with zeolite and MC shows higher paraquat desorption values compared to the hydrogel with zeolite and CMC.

In the analysis of the desorption properties of hydrogels with MC, studies were conducted on the release of the herbicide difenzoquat. The kinetic curves presented in Figure 4j–l show that all three samples exhibited a slower and more gradual desorption compared to other release mechanisms observed in this study across the three pH ranges. Notably, all samples demonstrated a gradual desorption for up to 24 h, followed by a decrease in herbicide concentration in the medium. However, after this reduction, a gradual increase in the concentration of released difenzoquat was observed, persisting even with changes in the release medium’s pH.

The slower and more gradual desorption of difenzoquat compared to paraquat, as described earlier in the herbicide release results with samples containing CMC, is attributed to the fact the difenzoquat is a neutral molecule (no net electrical charge). The values in Table 3 indicate that the desorption capacity of the hydrogel with zeolite and MC increases with higher pH, likely due to increased herbicide exchange/diffusion with ions in the medium, as the formal charge of the material’s polymeric chains rises with pH elevation. However, this phenomenon had the opposite effect on the hydrogel with MC, retaining a higher amount of herbicide molecules in its chains. In this case, the increased electrical charge led to stronger attractive forces between the herbicide and the hydrogel, hindering the transport of these molecules from the material’s interior to the release medium.

Comparing the difenzoquat release results of the hydrogels with MC (presented in Table 3) with the desorption values of the hydrogels with CMC, it is evident that the *q_eq_* values for the hydrogel with CMC were higher across all three pH ranges. This higher release of difenzoquat is due to the contribution of the carboxyl groups of CMC facilitating the diffusion of Na^+^ ions. However, the nanostructured hydrogel with MC obtained higher *q_eq_* values for difenzoquat compared to the nanostructured hydrogel with CMC. This was attributed to the zeolite increasing the attractive forces between its chains and the herbicide molecules rather than favoring exchange/diffusion as observed in the case of the hydrogel with zeolite and MC. The polymeric chains of the nanostructured hydrogel with CMC captured a larger amount of difenzoquat molecules in its structure. The findings suggest that the pH of the release medium plays a significant role in the controlled release of herbicides from the hydrogel systems.

The amount of herbicide adsorbed and/or released was higher than what has been reported by Filho et al. [31], where hydrogels composed of pectin and montmorillonite were used to remove the herbicide 2,4-dichlorophenoxyacetic acid, achieving removal rates of less than 2 mg·g^−1^. As for desorption properties, these matrices released a significant amount only in hydrochloric acid and ethanol over a 24 h period. In comparison to previous results obtained by the authors [11,26], with the herbicide diquat, a similar profile was observed for paraquat. However, the release of difenzoquat in both samples occurred more gradually and in a more controlled manner, possibly due to its higher molecular weight and lower formal charge compared to diquat.

## 3. Materials and Methods

### 3.1. Materials

The materials used and their respective suppliers are listed in Table 4.

### 3.2. Nanocomposite Hydrogel Synthesis

Hydrogel and hydrogel nanocomposites based on cellulose derivatives and zeolite were obtained using the same synthesis process described by the authors in previous works [9,11,26]. The synthesis of hydrogels and nanocomposite hydrogels was performed by dissolving the reagents (zeolite, CMC or MC, monomers (5.0% *w*/*v* of MAA and 5.0% *w*/*v* of AAm), and crosslinker MBAAm (3 mol% in relation to monomers)) in an aqueous medium, followed by introducing a nitrogen atmosphere and the initiator K_2_S_2_O_8_ (C_final_ = 6.17 mmol·L^−1^). Once all the components were incorporated, the solution was placed in an acrylic mold and heated in an oven at 70 °C for 24 h.

**Scheme 1:**
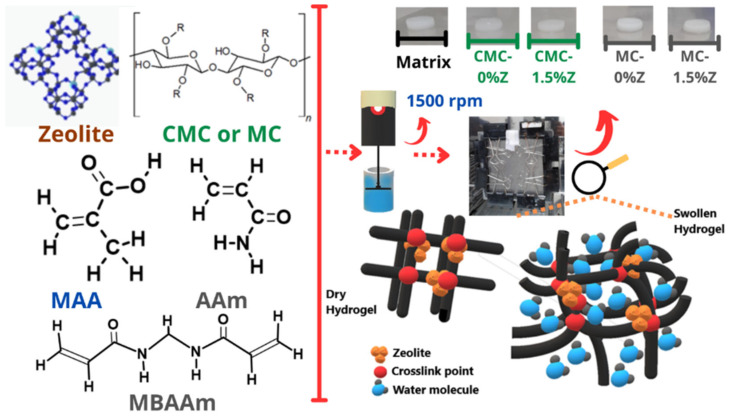
PMAA-co-PAAm hydrogel nanocomposites with cellulose derivatives (CMC or MC) and zeolite synthesis process illustration.

Scheme 1 illustrates the schematic image of the synthesis process.

For the swelling, sorption, and desorption studies, samples were obtained using a rubber spacer with a diameter of 0.5 cm. The samples used in the thermal analysis were prepared by grinding the samples into powder form. All samples were dried at a controlled temperature of 40 °C for 24 h. For characterization, the hybrid matrices were labeled according to the notation X-Y, where X represents the polysaccharide used in the synthesis (0.75% *w*/*v* of CMC or 1.0% *w*/*v* of MC), and Y denotes the content of zeolite (0–1.5%) in the hydrogel structure.

### 3.3. Nanocomposite Hydrogel Characterization Methods

#### 3.3.1. Apparent Viscosity (μ)

To study the stability of zeolite in the polymerization solution induced by the thickening effect of cellulose derivatives CMC and MC, measurements of apparent viscosity were conducted at 25 ± 1 °C using a Brookfield DV-II + Pro viscometer (Middleboro, MA, USA) for the hydrogels and their respective nanocomposites containing 1.5% zeolite. This concentration used in the viscosity analyses was chosen as the maximum concentration that the amount of CMC and MC could stabilize the zeolite without the formation of precipitates. The pH of the solutions was also determined using a GEHAKA pH meter (Sao Paulo, Brazil). This investigation can provide insights into potential interactions that contribute to a more homogeneous distribution of zeolite in the solution and the material structure.

#### 3.3.2. Thermogravimetric Analysis (TG)

The thermal stability of several matrices was investigated using thermogravimetric analysis (TG). The measurements were conducted under an inert nitrogen gas atmosphere with a flow rate of 100 mL·min^−1^. The heating process ranged from room temperature to 800 °C at a rate of 10 °C·min^−1^. The analyses were performed using a TA Instruments SDT Q600 instrument (New Castle, DE, USA), with approximately 8–10 mg of dried powdered samples placed in a platinum sample holder.

#### 3.3.3. Degree of Swelling (SD)

The swelling degree (*SD_t_*) values, measured at 25 ± 1 °C, were calculated using Equation (2), where *m_t_* represents the sample mass after immersion in the swelling solution for time ‘t’ and *m_d_* denotes the dried sample mass.

(2) $${SD}_{t}=\frac{m_{t}}{m_{d}}$$

The swelling equilibrium state (*SD_eq_*) represents the point at which the hydrogel reaches its maximum capacity for water absorption within its structure. To capture this state, values were chosen from the 48 h swelling period. For the investigation of the ionic effect, saline solutions with specific concentrations were prepared: NaCl (0.05, 0.10, 0.15, and 0.20 mol·L^−1^), CaCl_2_ (0.15 mol·L^−1^), or AlCl_3_ (0.15 mol·L^−1^).

#### 3.3.4. Herbicide Sorption and Release Properties

The potential use of these nanocomposites for agricultural applications, such as in water remediation or carrier vehicles in controlled-release systems for herbicides, was assessed through sorption and desorption analyses using UV-visible techniques. Sorption measurements were performed by immersing dried samples (between 0.08 and 0.1 g) of the hydrogel in 20 mL of herbicide solutions with a concentration of 40 mg·L^−1^ (λ = 257 nm for paraquat (PQ), R^2^ = 0.9994, and λ = 254 nm for difenzoquat (DFQ), R^2^ = 0.9996). Herbicide quantification was determined using a Shimadzu UV-VIS-2600 UV/VIS spectrophotometer (Kyoto, Japan) [10].

The desorption studies were conducted by placing the herbicide-loaded samples into 30 mL of 0.1 mol·L^−1^ NaCl solutions at pH 4, 7, or 10. These analyses were performed to investigate the influence of pH on the release properties, and they were necessary because herbicide desorption was not observed when deionized water was used as the release medium. The amount of PQ or DFQ loaded or released per gram of hydrogels (*q_t_*) was calculated using Equation (3).

(3) $$q_{t}=\frac{\left[C_{0}-C_{t}\right]\times V}{W}$$

where *C_t_* and *C*_0_ represent the concentration of PQ or DFQ in the solution at time ‘*t*’ during the sorption/desorption process and the initial amount or concentration of the herbicide loaded by the hydrogel in the desorption analyses, respectively. The volume of the sorption/desorption solutions and the weight of the hydrogel used in the measurements are represented by *V* and *W*, respectively.

Sorption and desorption analyses were all performed at room temperature (25 ± 1 °C) at predetermined time intervals (1, 2, 4, 8, 24, 30, and 48 h).

The percentage of herbicide adsorbed and the cumulative release (CR) of PQ or DFQ at different time intervals were calculated using Equations (4) and (5), respectively.

% adsorbed of herbicide = (C_t_/C_0_) × 100 (4)

CR (%) = (CA_t_/AL_∝_) × 100 (5)

where CA_t_ represents the cumulative amount of herbicide released at time ‘t’, and AL_α_ represents the amount of herbicide loaded into the hydrogel.

## 4. Conclusions

This study investigated the incorporation of zeolite into hydrogels based on cellulose derivatives (CMC and MC) and PMAA-co-PAAm networks for potential agricultural applications in controlled herbicide release. The results revealed significant improvements in the stability and thermal properties of the nanocomposite hydrogels with zeolite. The apparent viscosity measurements showed that the addition of 0.75% *w*/*v* of CMC and 1.0% *w*/*v* of MC increased the viscosity of the medium and contributed to stabilizing zeolite during synthesis, preventing precipitate formation. Moreover, the incorporation of zeolite in the hydrogel matrix enhanced thermal stability, as demonstrated by the TG and DTG.

Swelling degree experiments indicated that the nanocomposite hydrogels showed superior performance compared to the hydrogel matrix in solutions containing bi- and trivalent salts, indicating a lower influence of external forces on their atomic and molecular interactions.

Herbicide sorption and release studies revealed that hydrogels with CMC demonstrated higher herbicide adsorption capacity and faster release compared to hydrogels with MC. The presence of zeolite in the hydrogel enhanced the adsorptive capacity for both herbicides, particularly difenzoquat, surpassing the performance of the hydrogel matrix. The controlled release of herbicides from the hydrogel systems was strongly influenced by the type of hydrogel and the pH of the release medium.

Overall, the results suggest that nanostructured hydrogels with zeolite have great potential for controlled herbicide release in agriculture, and that their properties can be tailored through the zeolite presence and the selection of cellulose derivatives. This research opens new possibilities for the development of sustainable and environmentally friendly agricultural practices, contributing to the reduction in herbicide application and its negative impact on the ecosystem.

## Figures and Tables

**Table 1:** Viscosity values of polymeric solutions of hydrogels and nanostructured hydrogels with MC and CMC.

Solution	Viscosity (cP)
Water	1.0
Hydrogel CMC	336.9
Nanocomposite CMC-1.5% zeolite	341.1
Hydrogel MC	41.2
Nanocomposite MC-1.5% zeolite	38.2

**Table 2:** Values of initial (T_0_), final (T_f_), and maximum (T_max_) temperature, and the % weight of the first, second, and third peaks observed in the DTG curves of the MC-containing hydrogels.

0% Zeolite				
	T_0_ (°C)	T_f_ (°C)	T_max_ (°C)	% Weigh
1° event	38.7	163.2	114.0	5.4
2° event	163.2	271.7	205.5	12.6
3° event	271.7	556.5	349.4	67.5
0.5% Zeolite				
	T_0_ (°C)	T_f_ (°C)	T_max_ (°C)	% Weigh
1° event	38.0	170.1	91.5	6.1
2° event	170.1	275.9	207.0	12.3
3° event	275.9	582.8	375.6	67.9
1.0% Zeolite				
	T_0_ (°C)	T_f_ (°C)	T_max_ (°C)	% Weigh
1° event	30.9	165.8	52.8	7.4
2° event	165.8	275.2	208.1	11.8
3° event	275.2	598.5	352.3	66.7
1.5% Zeolite				
	T_0_ (°C)	T_f_ (°C)	T_max_ (°C)	% Weigh
1° event	35.1	175.1	57.3	6.6
2° event	175.1	275.2	207.8	10.6
3° event	275.2	599.2	377.7	61.5

**Table 3:** Released concentration values, amount of paraquat or difenzoquat (mg) released per gram of hydrogel, and percentage of herbicide (paraquat or difenzoquat) released in the desorption medium by the matrix hydrogel and hydrogel nanocomposite with MC and CMC at different pH ranges (4, 7, and 10).

	PQ			
	pH	C_eq_ (mg·L^−1^)	%L_eq_	*q_eq_* (mg·g^−1^)
	4	15.35	40.76	6.12
Matrix	7	14.17	37.55	4.94
	10	12.40	32.83	4.26
	4	14.31	37.75	4.46
CMC-0% Z	7	14.74	37.40	4.96
	10	14.32	36.31	4.53
	4	10.68	27.24	3.23
CMC-1.5% Z	7	10.47	26.57	3.17
	10	11.62	29.50	3.80
	4	13.71	34.90	3.07
MC-0% Z	7	14.73	37.46	3.20
	10	13.44	34.12	3.07
	4	12.91	32.75	4.94
MC-1.5%	7	10.44	26.46	4.01
	10	12.07	30.61	4.00
	DFZ			
	pH	C_eq_ (mg·L^−1^)	%L_eq_	*q_eq_* (mg·g^−1^)
	4	14.84	31.10	4.76
Matrix	7	16.00	35.06	5.73
	10	16.43	33.80	6.01
	4	15.80	32.81	6.52
CMC-0% Z	7	18.30	38.00	7.22
	10	19.00	39.00	7.12
	4	17.93	37.70	6.52
CMC-1.5% Z	7	17.44	36.00	4.53
	10	19.46	40.16	7.00
	4	16.45	34.20	5.00
MC-0% Z	7	16.74	34.63	4.90
	10	16.91	34.80	4.86
MC-1.5% Z	4	18.23	37.92	6.66
	7	17.10	35.34	5.20
	10	20.00	41.12	8.06

**Table 4:** Materials and providers of the reagents used in the synthesis and analyses of the nanocomposites and hydrogels studied in this work.

Materials	Providers
Methacrylic acid (MAA—99%)	Aldrich (St. Louis, MO, USA)
Acrylamide (AAm—98%)	Vetec-Brazil (Duque de Caxias, Brazil)
N ‘, N methylenebisacrylamide (MBAAm—98%)	Vetec-Brasil (Duque de Caxias, Brazil)
Potassium persulfate (K_2_S_2_O_8_—≥99%)	Sigma (St. Louis, MO, USA)
Carboxymethylcellulose (CMC, M_v_ = 114 × 10^3^ g/mol)	Vetec-Brasil (Duque de Caxias, Brazil)
Methylcellulose (MC, M_w_ = 40 × 10^3^ g/mol)	Sigma (St. Louis, MO, USA)
Zeolite clinoptilolite ZK406H (porous size 4–7 Å, cation exchange capacity (CEC) 0.8–1.2 meq·g^−1^, surface charge density 10.1 × 10^−23^ meq/Å^2^, and 99% purity)	St. Cloud Minning (Winston, NM, USA)
Paraquat hydrated dichloride (99.5%)	Chem Service (West Chester, PA, USA)
Difenzoquat methyl sulfate (99.5%)	Chem Service (West Chester, PA, USA)
Sodium chloride (NaCl, 99%)	Vetec-Brazil (Duque de Caxias, Brazil)
Calcium chloride (CaCl_2_, 96%)	Vetec-Brazil (Duque de Caxias, Brazil)
Aluminum chloride (AlCl_3_, 98%)	Vetec-Brazil (Duque de Caxias, Brazil)
Sodium hydroxide (NaOH, ≥95%)	Vetec-Brazil (Duque de Caxias, Brazil)
Hydrochloric acid (HCl, 36.5–38%)	Synth-Brazil (Sao Paulo, Brazil)

## Data Availability

Data can be made available upon request to the corresponding author.
